# Primary Open Angle Glaucoma is Associated with MR Biomarkers of Cerebral Small Vessel Disease

**DOI:** 10.1038/srep22160

**Published:** 2016-02-29

**Authors:** Karl Mercieca, John Cain, Thomas Hansen, Laura Steeples, Amy Watkins, Fiona Spencer, Alan Jackson

**Affiliations:** 1 Manchester Royal Eye Hospital, Oxford Road, Manchester, M13 9WL, UK; 2 Wolfson Molecular Imaging Centre, University of Manchester, 27 Palatine Road, Withington, Manchester, M20 3LJ, UK

## Abstract

This prospective study tests the hypotheses that: 1) glaucoma is associated with evidence of cerebral small vessel disease; 2) that imaging biomarkers of cerebral small vessel disease in POAG and NTG will show different characteristics. 12 normal controls, 7 patients with primary open angle glaucoma (POAG) and 9 patients with normal tension glaucoma (NTG) were recruited. Ophthalmological clinical assessment and MR imaging of the brain were performed. MR imaging was used to quantify white matter lesion load, frequency of dilated perivascular spaces (PVS) and abnormalities in cerebral hydrodynamics. Patients with POAG had significantly greater white matter lesion load (p < 0.05), more PVS in the centrum semiovale (p < 0.05) and had higher overall PVS scores than controls (p < 0.05). In the POAG group, optic cup-to-disc ratio (CDR) was positively correlated with deep white matter hyperintensities (R^2^ = 0.928, p < 0.01). Mean deviation on the Humphrey visual field assessment was negatively correlated with deep white matter lesion load (R^2^ = −0.840, p < 0.01), total white matter lesion load (R^2^ = −0.928, p < 0.01) and total PVS (R^2^ = −0.820, p < 0.01). MR evidence of cerebral small vessel disease is strongly associated with a diagnosis of POAG and with the severity of abnormalities in CDR and visual field.

Glaucoma describes a group of optic neuropathies, characterised by slow progressive degeneration of retinal ganglion cells and their axons. This results in a characteristic appearance of the optic disc and progressive visual loss. Patients with glaucoma may show elevation of intra-ocular pressure (IOP) although in others the IOP is within normal limits Traditionally the term primary open and glaucoma (POAG) has been used to describe glaucoma associated with elevated IOP and treatment is directed towards normalisation of the pressure. However, apparently elevated IOP is relatively common in the normal population, many patients who fulfil the other clinical criteria of POAG have normal IOP and in many cases, glaucoma continues to progress, despite maintaining target IOP^1^. The term normal tension glaucoma (NTG) has been used to describe glaucoma where the IOP is within normal limits. However, there is considerable controversy in the literature over whether these disorders truly represent separate entities or simply extreme ends of a single distribution^2^. These entities do appear to differ in other ways, with different patterns and progression of field defects and the identification of risk factors such as migraine and nocturnal systemic hypotension in NTG^3^.

Recent research has supported the hypothesis that the relationship between IOP and CSF pressure may be of importance in glaucoma since it will directly affect the pressure gradient across the lamina cribrosa^3^,^4^,^5^,^6^. Previous experimental studies have shown that some patients with POAG have abnormally low cerebrospinal fluid pressure^3^ but identified significant overlap between normal and glaucomatous groups, suggesting that additional factors, other than trans-laminar pressure gradient, must play a factor. It is clear that there are factors other than IOP involved in the pathogenesis of POAG which can impact the apoptotic process^7^. Factors that have been implicated include low ocular perfusion pressure, reduced ocular blood flow, low systolic blood pressure, cardiovascular disease, migraine, smoking and vasospasm disorders^8^,^9^,^10^. The identification of multiple vascular risk factors in association with glaucoma has led some authors to study the relationship between glaucoma and cerebral small vessel disease. A previous study by Stroman *et al*. found larger numbers of white-matter lesions in patients with NTG than controls^11^ and a recent study demonstrated that intraocular pressure was higher in normal individuals with evidence of cerebral white-matter lesions^12^.

This is a preliminary hypothesis generating study that asks: 1) is glaucoma associated with evidence of cerebral small vessel disease; 2) do imaging biomarkers of cerebral small vessel disease in POAG and NTG show different characteristics.

## Methods

### Patients and Controls

Ethics approval was obtained from the National Research Ethics Committee North West (REC reference 11/H1011/5) and all subjects gave informed consent prior to inclusion. The study was carried out in accordance with the approved guidelines and in keeping with the submission approved by the ethics committee.

The study was a prospective, randomised, double masked controlled clinical pilot study. Participants were recruited from glaucoma and general clinics at Manchester Royal Eye Hospital, UK. Patients with a diagnosis of POAG and NTG were identified from case notes and an experienced glaucoma consultant (AFS) reviewed the diagnosis. Participants for the control group were recruited by poster advertisement in the Acute Referral Clinic (ARC) where people attending for minor non-glaucoma related eye problems and/or their relatives were invited to participate. Each participant was given one week to consider participation in the study.

Participants were assigned to three groups: POAG patients; NTG patients and non-glaucomatous controls controversy. All diagnoses were verified by an experienced consultant working within a major tertiary referral glaucoma service. Each NTG and POAG patient had both significant optic disc and typical visual field changes associated with glaucomatous optic nerve damage. Apart from a cup-disc ratio (CDR) of 0.6 or more, each glaucoma patient had to have significant changes associated with glaucoma such as visible loss of retinal ganglion cell layer, thinning of neuro-retinal rim, typical peri-papillary atrophy, disc hemorrhages, bayonetting and/or notching. A mean deviation (MD) of less than or equal to −6 dB was chosen as the limit for glaucomatous field defects. In view of the controversy over the existence of POAG and NTG as separate entities, we chose a wide cut off for presenting IOPs between the two groups – the presenting IOP in the NTG group was taken to be 18 mmHg or less; in the POAG group it was 26 mmHg or more.

All normal subjects had a comprehensive ophthalmic examination by a senior ophthalmologist to exclude any of the above clinical signs associated with glaucoma. IOP cut off for this group was taken as 19 mmHg. Formal visual fields testing was undertaken (using a Humphreys HFA II-i analyser (Carl Zeiss Meditec AG, Jena, Germany) and had to be completely within normal limits (refer to Table 1 for comparison of mean deviations and CDR between all groups).

Clinical data included: patient age, central corneal thickness (CCT), IOP at presentation, IOP on treatment, Humphrey visual field (HVF) assessment (specifically Mean Deviation (MD)) and optic disc assessment including CDR. Each participant’s group status was subsequently anonymized and subjects were referred for MRI scanning.

### MR Imaging

Scanning was performed on a 1.5 Tesla whole body scanner (Philips Acheiva, Philips Medical Systems, NL) using an 8-channel phased array head coil. The MR protocol consisted of 1) T1 inversion recovery (axial, TR/TE/TI −4572/15/400 ms, slice thickness 2 mm); 2) T2 fluid attenuated inversion recovery (axial, TR/TE/TI −6000/120/2000 ms, slice thickness 2 mm); 3) Survey phase contrast angiography to locate vessels for flow analysis (TR/TE −20/6.4 ms); 4) Quantitative Phase contrast imaging (QPC) of the carotid and basilar arteries (velocity encodation (VENC) 120 cm/s) and cerebral aqueduct (VENC 8 cm/s). 6) Axial SPIR FLAIR image of the orbit (TR/TE/TI −8000/120/2200 ms); 7) Coronal T2 SPIR of the orbit (TR/TE −3085/120 ms, slice thickness 2 mm).

### Image Analysis

#### Assessment of small vessel disease (SVD) Biomarkers

**Figure 1:**
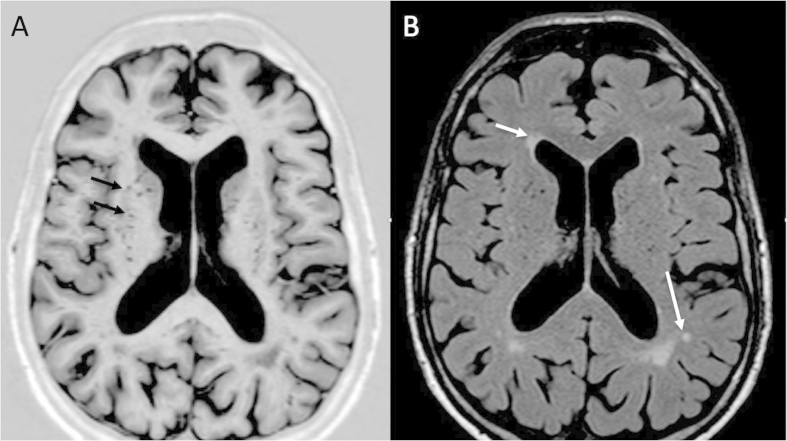
(**a**) T1 inversion recovery MRI of patient with POAG with multiple dilated PVS in the basal ganglia (black arrows), (**b**) Axial FLAIR MRI of same patient with periventricular white matter hyperintensities (white arrow) and small deep white matter hyperintensities (long white arrow).

Figure 1 shows example images illustrating dilated perivascular spaces (PVS) and white matter abnormalities. The severity of white matter abnormalities was assessed on FLAIR images using a modified version of Schelten’s score which has been described previously and validated in our laboratory^13^,^14^. Periventricular hyperintensity in the frontal, parietal, and occipital regions was scored: 0 for none, 1 = <5 mm thick, 2 = >5 mm thick. Abnormalities in the deep white matter (frontal, parietal, temporal, occipital), basal ganglia (lentiform and caudate nuclei, thalamus, internal capsule), and brainstem (cerebellar WM, midbrain and pontine reticular formations, medulla) were scored: 0 for none; 1 = five or fewer <3 mm, 2 = six or more <3 mm, 3 = five or fewer 4–10 mm, 4 = six or more 4–10 mm, 5 = >11 mm, and 6 for confluent (total scores of 0–24).

Dilatation of perivascular spaces (PVS) was assessed on Axial T1 inversion recovery images using a scoring system was adapted from Doubal *et al*. 2010^15^ as described previously^13^. The basal ganglia and centrum semiovale (CSOV) were scored separately and then the scores were combined to produce a total score. In each structure the slice with most dilated PVS visible was selected, and the number counted on the side of the brain with the greatest number. Scores were as follows; 0 = no PVS, 1 = 1–10, 2 = 11–20, 3 = 21–30, 4 = 31–40, and so on. The original Doubal score had 3 = 21–40, and 4 (>40 PVS) was the maximum score.

#### Assessment of Cerebral Hydrodynamics

Cerebral blood flow was measured using QPC images of the carotid and basilar arteries. Measurements were performed using Segment (Einar Heiberg^16^); regions of interest (ROIs) were drawn over each vessel lumen and flow rates were calculated for each of 16-gated measurements over the cardiac cycle. Total blood flow in each vessel was measured as the integral of the flow curve multiplied by the heart rate. CSF flow in the cerebral aqueduct was calculated using the same process. Arterial and CSF flow profiles were then entered into a locally developed software package developed in Interactive Data Language (IDL; Excelis Visual Information Solutions, VA, USA) which has been described previously^17^. This allows calculation of cerebral aqueductal systolic peak width, arterial systolic peak width, arterial aqueductal delay, and systolic and diastolic stroke volumes in the cerebral aqueduct.

#### Statistical Analysis

Statistical analysis was performed in SPSS 22 (IBM, USA). Between group differences were assessed using analysis of variance (ANOVA) with post hoc tests for between group differences assuming unequal variance. Correlation between clinical and imaging parameters was assessed using spearman’s correlation coefficient (rho) for non-parametric variables.

A data model was constructed to assess the contribution of imaging features to the separation of Norm, POAG and NTG groups. Data was standardised to produce z scores. Multinomial logistic regression modelling was performed in Wizard Pro (http://wizard.evanmiller.org/) treating diagnosis as the outcome class (1 = Norm, 2 = POAG and 3 = NTG). Imaging biomarker scores were entered as covariates in the model if they showed a correlation with diagnosis at significance less than 0.1. Variables were then sequentially removed from the model based on the significance of their contribution to the model until all remaining variables were significant at the p < 0.05 level. Patient age was entered as a covariate in the model.

## Results

One patient in the POAG group was unable to tolerate the MR examination and was excluded from the study. The final study group consisted of 12 Norm, 7 POAG and 9 NTG. There was no significant difference in the age or gender distribution between groups. The ophthalmological and demographic features are shown in Table 1.

**Figure 2:**
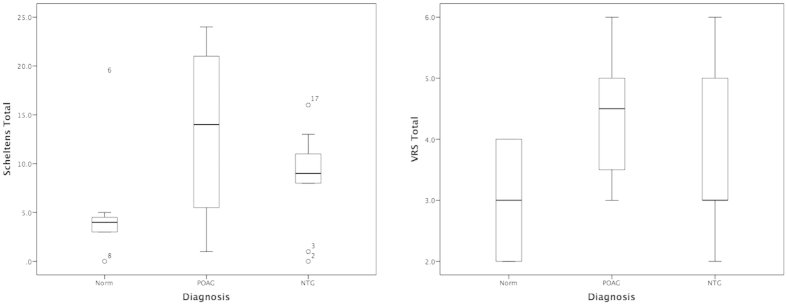
Boxplots showing a comparison of Schelten’s total scores and VRS total scores for all three groups (as described in text).

Patients with POAG showed significantly greater white matter lesion load (p < 0.05), more PVS in the CSOV (p < 0.05) and had higher overall PVS scores than controls (p < 0.05, Fig. 2). Patients with NTG had significantly shorter arterial systolic peak width than either normal (p < 0.01) or patients with POAG (p < 0.05). Patients with NTG had a trend towards decreased total cerebral blood flow, decreased aqueductal stroke volume and decreased arterial aqueductal delay compared to controls and patients with POAG although these failed to reach statistical significance.

In the group as a whole there were significant correlations between intra-ocular pressure and arterial systolic peak width (R^2^ = −0.716, p < 0.01). CDR was positively correlated with white matter lesion load (R^2^ = 0.518, p < 0.01), total PVS (R^2^ = 0.550, p < 0.01) and inversely correlated with total cerebral blood flow (R^2^ = −0.520, p < 0.01). Mean deviation on the Humphrey visual field assessment was positively correlated with basal ganglia PVS (R^2^ = 0.601, p < 0.01) and total PVS (R^2^ = 0.527, p < 0.01) and negatively correlated with white matter lesion load (R^2^ = −0.552, p < 0.01).

In the POAG group CDR was positively correlated with deep white matter hyperintensities (R^2^ = 0.928, p < 0.01). Mean deviation on the Humphrey visual field assessment was negatively correlated with deep white matter lesion load (R^2^ = −0.840, p < 0.01), total white matter lesion load (R^2^ = −0.928, p < 0.01) and total PVS (R^2^ = −0.820, p < 0.01).

In the NTG group no significant correlations were observed between clinical and imaging features.

Multinomial logistic regression modelling demonstrated basal ganglia PVS (Z score −2.27, p < 0.05), arterial aqueductal delay (Z score −2.01, p < 0.05) and total white matter lesion load (Z score 3.497, p < 0.05) as the only independently significant discriminators from the imaging variables. The overall model was significantly predictive of group membership (p < 0.05, X^2^ = 8.65) and the area under the receiver operator curve was 0.76.

## Discussion

Over the past 15 years there has been increasing recognition that elevation of intraocular pressure In POAG appears to represent one component in a multifactorial disease process rather than the primary pathogenic feature^5^,^6^,^18^,^19^. The identification of increased pressure gradients across the lamina cribrosa as a pathogenetic mechanism has received extensive support with the demonstration of increased gradients, reflecting decreased intra-cranial CSF pressure in both POAG and NTG. However some groups have failed to identify low CSF pressure in association with glaucoma and others have shown a significant overlap in CSF pressure between glaucomatous and normal individuals^3^,^18^. Other aetiological factors have been identified and, in particular, there appears to be a relationship between POAG and vascular risk factors which are hypothesized to result in reduced ocular perfusion^8^,^9^,^10^.

The existence of arteriosclerotic changes in small cerebral arteries, known as microvascular angiopathy, has been recognised for many years. The introduction of MRI in the late 1970s rapidly led to the realization that high signal white matter abnormalities are commonly seen, increasing in prevalence with age. These deep white matter hyperintensities have been attributed to ischaemic injury and have been widely used as a biomarker of cerebral small vessel disease (SVD)^20^. However, a histological study of patients with AD showed that of 87 patients with significant white matter abnormalities, small vessel disease was present in only 47^21^. These authors concluded that the aetiology of deep white matter hyper-intensities was likely to be multifactorial. This led other groups to try and identify biomarkers which had greater specificity for cerebral SVD^14^,^22^,^23^. From this work two main groups of biomarkers were identified. The first of these was the identification of dilated PVS in the basal ganglia and CSO^14^,^22^. PVS are a known histological feature of more severe grades of microvascular angiopathy and can be demonstrated on MR. Studies have shown a strong association between PVS and diseases related to the presence of SVD including vascular dementia^14^, treatment resistance in late-onset depression^22^,^24^ and high Framingham stroke risk index in normal individuals^25^. This led to many groups identifying PVS as a valuable indicator of the presence of SVD with greater specificity than white matter lesions^14^,^15^,^26^. In one recent large scale prospective study of 246 subjects PVS were associated with lacunar stroke subtype (p < 0.0001) age and white matter lesion load and these authors concluded that the findings were “consistent with the hypothesis that PVS reflect small vessel disease”^27^. A major consensus position paper published in Lancet Neurology, Wardlaw *et al*. identified PVS as an important biomarker of SVD. The presence of PVS has now been incorporated as a biomarker of SVD in many studies^28^,^29^,^30^,^31^,^32^,^33^,^34^,^35^,^36^,^38^. Dilated PVS can be seen as congenital lesions but their small number, typical distribution and unusual morphology make it impossible to mistake these for dilatations associated with small vessel disease. In addition dilation of PVS in the CSOV can be seen in severe cerebral atrophy although no such relationship has been reported with PVS in the basal ganglia. In the present study we showed significantly higher deep white matter lesion load and increased numbers of PVS in association with POAG suggesting the presence of significant small vessel disease. This finding is in keeping with the observations of Park *et al*.^12^ who recently demonstrated that high values of IOP were positively associated with the presence of deep white matter hyper intensities in middle-aged and elderly subjects without glaucoma. Other than this, to our knowledge, this is the first demonstration of a significant association between POAG and cerebral small vessel disease. Furthermore, there were strong relationships between mean deviation demonstrated on the Humphrey visual field assessment scale and both white-matter lesion load and PVS in patients with POAG. A similar relationship was seen between white matter lesion load and optic disc ratio.

The second group of imaging biomarkers uses quantitative measurements of arterial and CSF flow patterns. In the mid 1990s it was realized that the increased volume of blood in the cranial cavity during systole gives rise to a complex mechanism of CSF and venous blood displacement protects the brain from pulsatile trauma^39^. In the presence of arteriosclerosis this mechanism begins to fail leading to passage of the systolic pulse wave into brain tissue due to decreases in arteriolar compliance. Several studies have now shown that evidence of decreased compliance is seen in the presence of small vessel disease, in particular, decreased arterial compliance leads to more rapid passage of the pulse wave into the brain which is reflected by reduction in the arterial-aqueductal delay^17^. We consequently used these measurements in the current study but found no abnormalities in the POAG group.

Patients with NTG showed no significant differences in white matter lesion load or PVS compared to normal controls. However, they did have shorter arterial systolic peak width than either normal or POAG subjects. There was also a trend towards reduced cerebral blood flow and decreased aqueductal stroke volume in this group. Although these features did not reach significance when compared between groups, significant correlations between intra-ocular pressure and arterial systolic peak width and between optic disc ratio and total cerebral blood flow were seen when the groups were combined. This suggests that abnormalities in cerebral hydrodynamics may be significant in NTG or in the production of glaucoma associated features.

Multinomial regression modeling identified basal ganglia PVS, arterial aqueductal delay and white-matter lesion load as the only independently significant discriminators from the imaging variables. This reflects strong correlations between the various imaging biomarkers of small vessel disease and it is interesting that when these are eliminated arterial aqueductal delay becomes a significant predictive factor.

A major limitation of the current study was the small numbers of patients enrolled which may be reflected by the presence of trends towards relationships in the NTG group which failed to reach statistical significance. However, the study was planned to produce evidence to support further investigation of the stated hypotheses. Even within the small numbers it seems clear that there is extensive, significant evidence that POAG is strongly associated with the presence of cerebral small vessel disease. The results suggest possible abnormalities in the NTG group and justify performance of a larger study to validate and better understand these findings.

In conclusion we have presented a preliminary study to test two specific hypotheses. The hypothesis that glaucoma is associated with evidence of cerebral small vessel disease was strongly supported in the POAG group. The hypothesis that imaging biomarkers of cerebral SVD in POAG and NTG will show different characteristics was also strongly supported. In addition we have shown strong correlations between evidence of SVD, CDR and field defects in patients with POAG. Limitations related to study size restrict the conclusions that can be drawn concerning the pathogenetic mechanisms which may relate cerebral SVD and glaucoma but the findings strongly support the need for a large-scale prospective study of small vessel disease in glaucoma^5^,^6^,^18^.

## Additional Information

**How to cite this article**: Mercieca, K. *et al*. Primary Open Angle Glaucoma is Associated with MR Biomarkers of Cerebral Small Vessel Disease. *Sci. Rep.* **6**, 22160; doi: 10.1038/srep22160 (2016).

## Figures and Tables

**Table 1:** Demographic and clinical features of subjects in the three study groups.

	NTG	POAG	CONTROLS
Age (mean/range)	61.0/41–86	53.6/50–82	65.5/54–75
M/F ratio	4/5	4/3	6/6
Presenting IOP mmHg	Mean 16 (12–18)	Mean 35 (26–40)	N/A
Current IOP mmHg	Mean 12 (10–17)	Mean 16 (11–19)	Mean 15 (10–19)
CCT (microns)	524	531	569
CD ratio	Mean 0.7 (0.6–0.95)	Mean 0.75 (0.6–0.90)	Mean 0.3 (0.2–0.5)
Visual field MD	−12.33 db	−10.19 db	+0.41 db
Visual field PSD	12.41	9.72	1.27
